# Tellu – an object-detector algorithm for automatic classification of intestinal organoids

**DOI:** 10.1242/dmm.049756

**Published:** 2023-03-13

**Authors:** Eva Domènech-Moreno, Anders Brandt, Toni T. Lemmetyinen, Linnea Wartiovaara, Tomi P. Mäkelä, Saara Ollila

**Affiliations:** ^1^ HiLIFE-Helsinki Institute of Life Science, Yliopistonkatu 4, 00014 University of Helsinki, 00100 Helsinki, Finland; ^2^ iCAN Digital Precision Cancer Medicine Flagship, Stenbäckinkatu 9 Hallintokeskus, University of Helsinki, 00290 Helsinki, Finland; ^3^ Translational Cancer Medicine Program, University of Helsinki, 00100 Helsinki, Finland

**Keywords:** Automation, Classifier, Intestinal organoid, Object detection

## Abstract

Intestinal epithelial organoids recapitulate many of the *in vivo* features of the intestinal epithelium, thus representing excellent research models. Morphology of the organoids based on light-microscopy images is used as a proxy to assess the biological state of the intestinal epithelium. Currently, organoid classification is manual and, therefore, subjective and time consuming, hampering large-scale quantitative analyses. Here, we describe Tellu, an object–detector algorithm trained to classify cultured intestinal organoids. Tellu was trained by manual annotation of >20,000 intestinal organoids to identify cystic non-budding organoids, early organoids, late organoids and spheroids. Tellu can also be used to quantify the relative organoid size, and can classify intestinal organoids into these four subclasses with accuracy comparable to that of trained scientists but is significantly faster and without bias. Tellu is provided as an open, user-friendly online tool to benefit the increasing number of investigations using organoids through fast and unbiased organoid morphology and size analysis.

## Teaser

**Summary:** Description and demonstration of an automated easy-to-use online tool for classifying intestinal organoids to facilitate quantitative analyses of increasingly popular organoids.

## INTRODUCTION

The intestinal epithelium has been extensively studied in order to understand stem cell biology and several important diseases, including colorectal cancer. These studies have been accelerated by developing conditions that allow culturing of primary intestinal epithelium as 3D organoids *ex vivo* (Sato et al., 2009). The intestinal epithelial organoids are self-organising structures that, in many ways, recapitulate *in vivo* architecture of the intestinal epithelium, including crypt and villus units and growth factor requirements that resemble the *in vivo* niche-derived factors. Stem cells in these organoids recapitulate their *in vivo* behaviour, producing new stem cells as well as all differentiated cell types of the intestinal epithelium, thereby allowing for continuous passaging (Sato et al., 2011). During culture, organoids develop from cystic non-budding organoids enriched for stem and Paneth cells to early organoids with one to two crypt units and, further, to late organoids with three or more crypt units that surround the central part, representing the villus compartment that differentiates during culture. Under certain conditions, organoids form spheroids that represent a less differentiated state of the epithelium (Fordham et al., 2013).

Morphological classification and quantitative analysis of organoids have proved helpful in understanding stem cell biology and disease. For example, analysis of the organoid growth and differentiation stimulated by exogenous growth factors or other signalling molecules can be addressed by measuring the *de novo* crypt-budding reflecting stemness capacity (Almeqdadi et al., 2019). Analysis of spheroid formation has been useful in understanding differences between adult and foetal intestinal epithelium (Guiu et al., 2019), and in describing features of regenerating epithelium (Nusse et al., 2018). In addition, spheroid morphology is induced by WNT ligands (Farin et al., 2012), mutations in the tumour suppressor gene APC (Langlands et al., 2018) or co-culture with fibroblasts (see Fig. S4 in Farin et al., 2012), highlighting the impact of WNT signalling and stromal cells to epithelial differentiation. As the organoids grow in 3D, classification of their morphology requires time-consuming manual analysis, which is subjective, and prone to variations between experiments and bias. Artefacts absent in conventional 2D cell culture, such as collapsing, overlapping and out-of-focus organoids, complicate classic image processing and segmentation approaches, and make automated workflows difficult. More efficient quantification methods would expand the opportunities to use intestinal organoid classification in various research applications.

Although some tools and pipelines have been developed to quantitate organoids, they have certain limitations for classification. *OrgaQuant*, a deep convolutional neural network trained by Kassis et al. (2019), is limited to quantifying the size of intestinal spheroids and does not include morphological analyses or classification capabilities. Spiller and colleagues developed a machine learning-based tool to differentiate between live and dead organoids in order to facilitate drug screening in patient-derived organoids (Spiller et al., 2021). Although a potentially valuable tool for translational purposes, this method is limited to viability analyses and was not trained to classify organoids. In an interesting study on epigenetic control of intestinal epithelial differentiation, Ostrop and colleagues developed a pipeline to classify organoids (Ostrop et al., 2020). However, the requirement of a manual selection of features to train the classifier and the need to separately define classes for each experiment restricts the use of this pipeline as an easy-access method to enable automated classification of organoids.

In this work, we developed Tellu – an easy-to-use organoid classifier tool, i.e. a machine learning algorithm – and show that it enables fast, accurate and reproducible automated analysis of intestinal organoid morphology.

## RESULTS

**Fig. 1.**
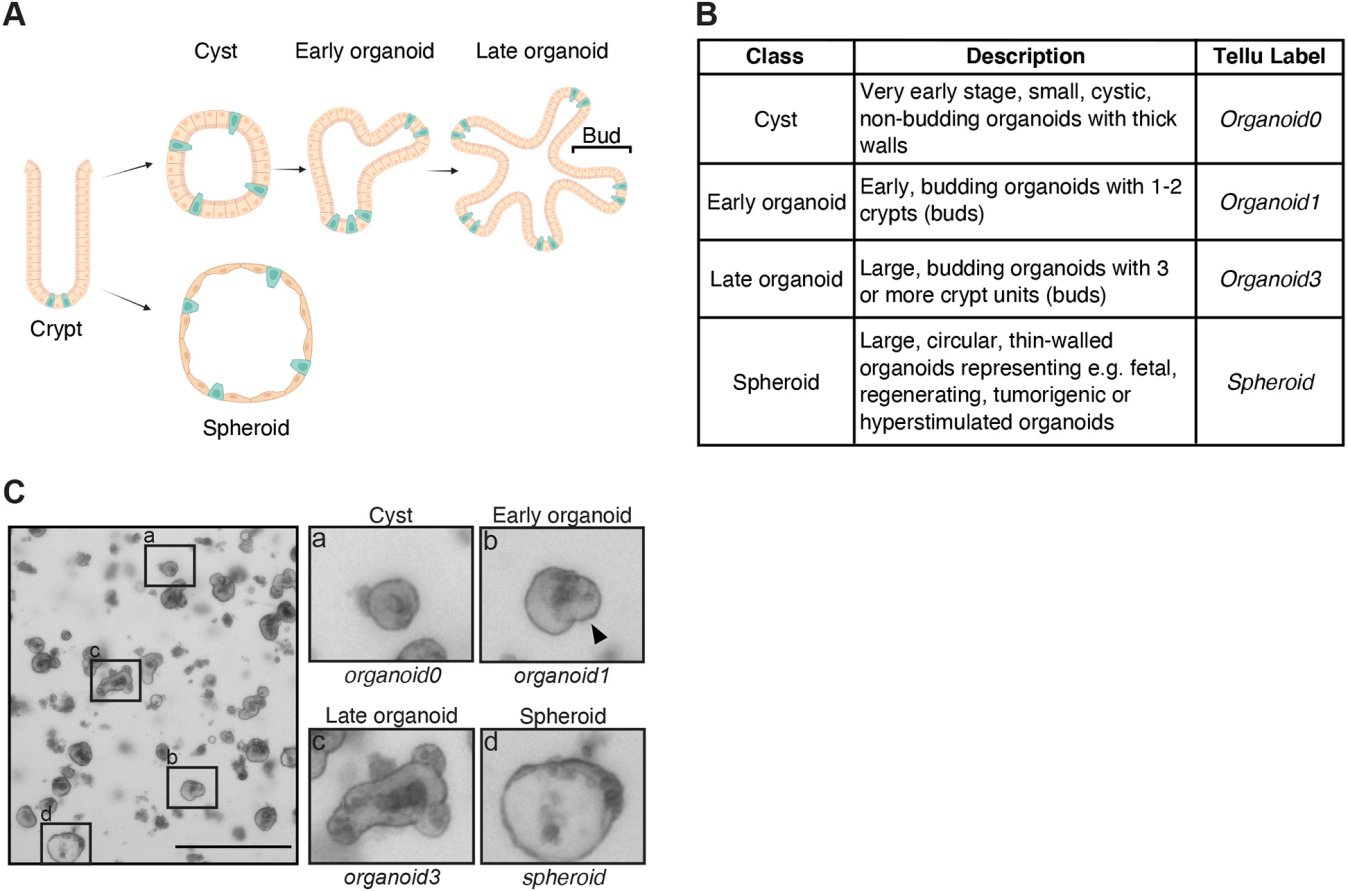
**Classification of intestinal organoids.** (A) Schematic depicting the formation of intestinal organoids from crypts isolated from the intestine and embedded in Matrigel. Embedded crypts initially form rounded cyst-like structures. Within the first days of culture, these cystic structures polarise (Serra et al., 2019) and develop into the crypt and villus domains, also known as buds (Early organoid). Typically during days 3-6, more crypt-budding events occur (Late organoid). Under certain conditions, isolated crypts do not develop into budding organoids but acquire a spheroid morphology (Spheroid). Green cells represent stem cells. Illustration created with BioRender.com. (B) Summary of organoid classes, their description and corresponding labels in Tellu output. (C) Representative image showing the four organoid classes; boxed areas are shown magnified (x3) on the right. The arrowhead indicates a developing crypt. Scale bar: 500 μm.

To develop a tool allowing automated classification of intestinal organoid morphology, we trained a real-time object detector algorithm. For this, we first created an annotated dataset of intestinal organoid images. Transmitted-light microscopy images of intestinal organoids derived from crypts isolated from the entire mouse small intestine at different stages of growth were manually divided into four classes of intestinal organoids based on their morphology. Non-budding, cystic organoids were labelled ‘*organoid0’,* early organoids with one or two crypt units ‘*organoid1’,* and late organoids with three or more buds ‘*organoid3’.* A separate class was created for ‘*spheroids’* that lack crypt structures, and display a thin wall and larger diameter than cystic non-budding organoids (Fig. 1). The total dataset consisted of 840 images with a total of 23,066 annotations. Each image contained an average of 28.2 annotations (median 21; range 1-137). Since all organoids initially start from a cystic shape (*organoid0*), the four classes in the dataset were not equally represented: *organoid0* represented 52% of the dataset with 11,922 instances, followed by *organoid1* (24%; 5510 instances), *organoid3* (14%; 3368 instances) and *spheroid* (10%; 2266 instances). We decided to split the annotated dataset into training (90%) versus model validation (10%) to maximize the size of the training dataset. Since this approach – instead of using the 80% versus 20% share that is frequently used – might render the validation set being too small to be representative, further validations were used to ensure that the model correctly generalises the organoid classes (see Figs 3–5).

### Developing a tool for automated detection and classification of intestinal organoids

**Fig. 2.**
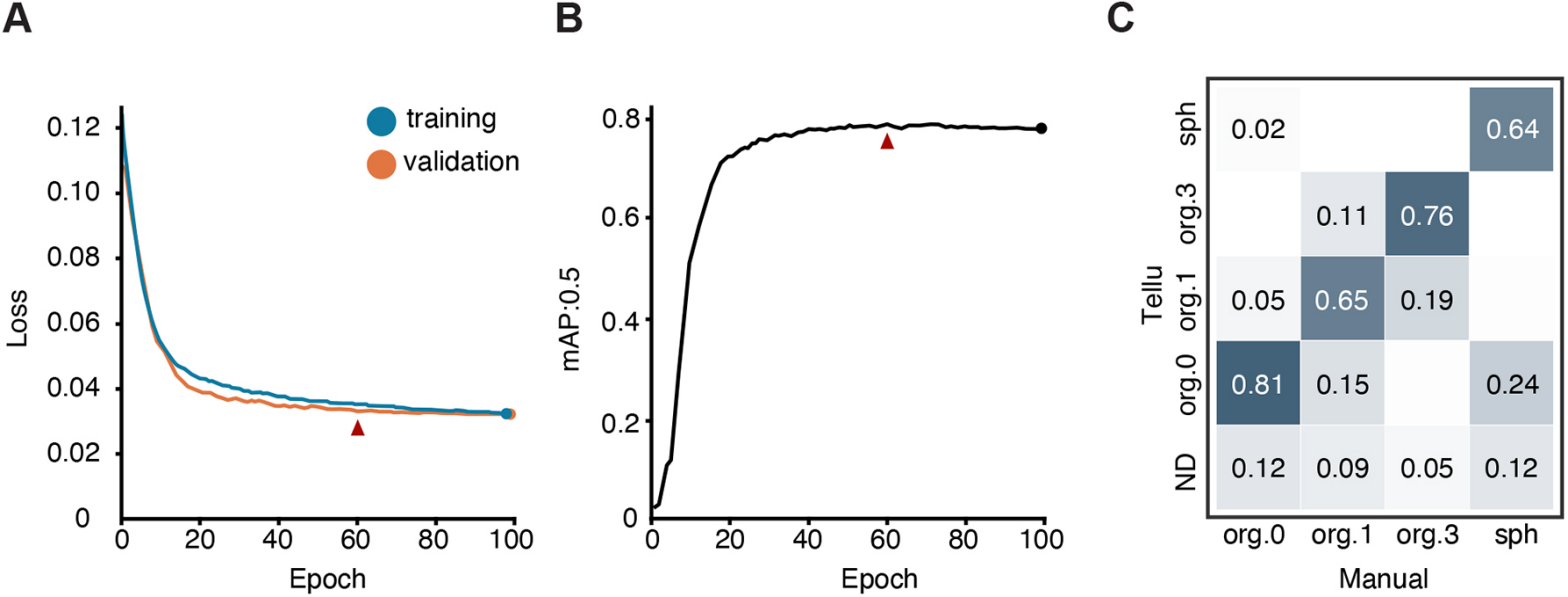
**Training and precision of organoid classification by Tellu.** (A) Model loss-of-function at each epoch for training (blue) and validation (orange) image sets. The arrowhead indicates the epoch (60) chosen as the endpoint for the learning. (B) Plotted is the mean average precision score of Tellu (mAP:0.5) across all organoid classes for each epoch. The arrowhead indicates the epoch (60) chosen as the endpoint for the learning. (C) Confusion matrix of organoids predicted by Tellu agast the ground truth (Manual). Labels indicate; cysts (org.0), early organoids (org.1), late organoids (org.3), spheroids (sph). ND, not detected.

To automate organoid classification, we chose the You Only Look Once (YOLO) object-detection algorithm that both predicts and classifies objects labelled as bounding boxes in an image (Redmon et al., 2016). We used a pre-trained YOLOv5 small model as the starting point and trained it by using a dataset of 756 images for 100 epochs (with one epoch going once through the whole dataset of images). Before reaching 100 epochs, the loss of function of the model for the training image set had converged and crossed the loss of function of the validation set, indicating that the learning rate slowed and that further iterations would likely lead the model to overfit, a process where the model keeps learning the training image set but fails to generalise the learning to data that has not seen before (Fig. 2A). To evaluate the model's performance, we assessed its precision (i.e. correctly detecting positive objects) and recall (i.e. not missing any objects) by using the mean average precision (mAP), a standard metric to evaluate overall model accuracy in the range of 0 to 1, with 1 representing an exact match to manually labelled detection. We used the mAP at a threshold of 50% overlap, i.e. at mAP=0.5, across the validation dataset, i.e. a set of 84 images, that was not used in training. The model achieved the best performance at epoch 60 (mAP:0.5=0.79) across classes (Fig. 2B); therefore, we chose it as the final learning point.

The classification performance of Tellu is shown as a confusion matrix displaying the proportion of the labelled dataset overlapping between ground truth – i.e. manual annotation by the trainer; *x*-axis – and the predicted classification – i.e. annotated by Tellu, *y*-axis (Fig. 2C). There, each column adds up to 1 and represents all annotations of that particular class. Performance in all classes was very good, with best performance for cysts (*organoid0;* 0.81) and late organoids (*organoid3;* 0.76). However, we noticed some differences in two situations: for early organoids, i.e. *organoid1*, Tellu labelled 0.15 and 0.11 of the annotations to *organoid0* and *organoid3*, respectively. The difference reflects the non-binary continuum from cysts (*organoid0*) *to late organoids* (*organoid3*), an ambiguity that is also seen between manual counters. There was also some difference in the distinction between spheroids and early cysts (*organoid0*), where morphological changes between these two classes at very early stages of culture are minimal. Here, Tellu labelled 0.24 of spheroids to cysts (*organoid0*). Failures of Tellu due to overlap or out-of-focus organoids were modest: 0.05 for *organoid3*, 0.09 for *organoid1,* and 0.12 for *organoid0* and *spheroid* (Fig. 2C).

### Intestinal organoid classification performance of Tellu

Next, we assessed the intestinal organoid classification performance of Tellu by comparing its classification to that of two scientists experienced in manual intestinal organoid classification under variable conditions.

**Fig. 3.**
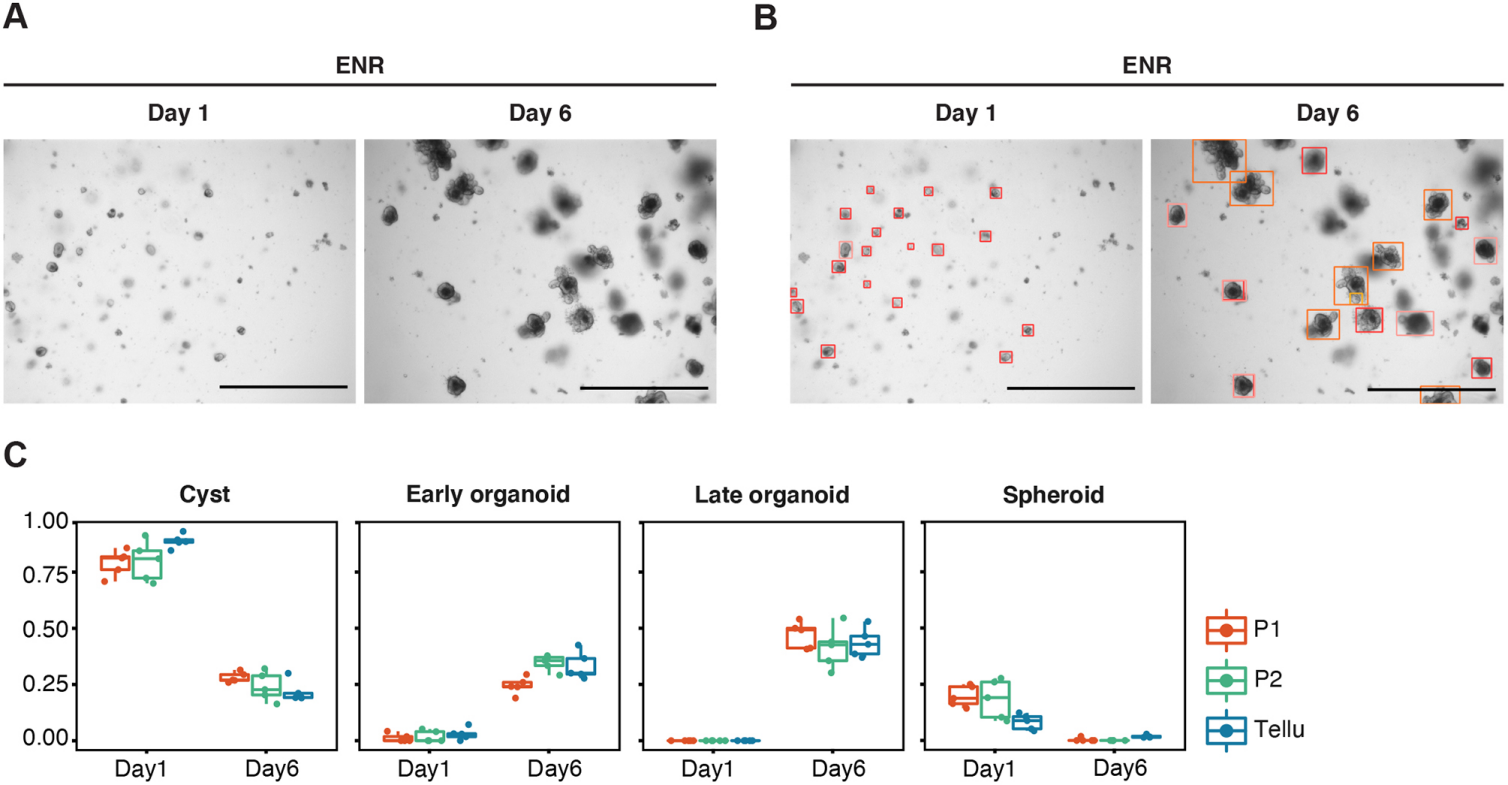
**Performance of Tellu regarding the classification of intestinal organoids at days 1 and 6 of normal growth compared to that of two experienced scientists.** (A) Representative images of organoids on days 1 and 6 that had been plated in Matrigel and cultured standard growth medium supplemented with EGF/Noggin/R-spondin1 (ENR). (B) Images as shown in A but including bounding boxes surrounding objects recognised by Tellu. Scale bars: 1 mm. (C) Compared is the classification of organoids on days 1 and 6, carried out by two experienced scientists (P1 and P2) or automated analysis (Tellu). Each data point represents a relative share of each organoid class per technical replicate (well) (*n*=270 organoids day 1, *n*=299 organoids day 6).

In the first experiment, classification performance was assessed at two time points (days 1 and 6) of normal intestinal organoid growth in the standard growth medium supplemented with EGF/Noggin/R-spondin1 (ENR) (Materials and Methods), with >200 organoids at either time point (Fig. 3). Remarkable alignment of the classifications for all classes was seen on day 6 [*cyst* (P1=0.28±0.02; P2=0.24±0.06; Tellu=0.21±0.04); *early organoid* (P1=0.24±0.04; P2=0.34±0.03; Tellu=0.33±0.06); *late organoid* (P1=0.47±0.05; P2=0.41±0.09; Tellu=0.43±0.06)]. Compared to both manual and automated analysis at day 1, reduction of cysts as well as induction of early and late organoids on day 6 demonstrates that Tellu faithfully captured the changes in organoid morphology. On day 1, when most organoids are at cyst stage (Fig. 2C, Cyst) and have a reduced ratio of spheroids (Fig. 2C, Spheroid), the Tellu classification was somewhat increased for cysts. However, this difference was only observed at very early time points. When we examined the detections made by Tellu, we noticed this disagreement was due to Tellu classifying single cells as cysts, a fact most probably derived from a harsh crypt dissociation.

**Fig. 4.**
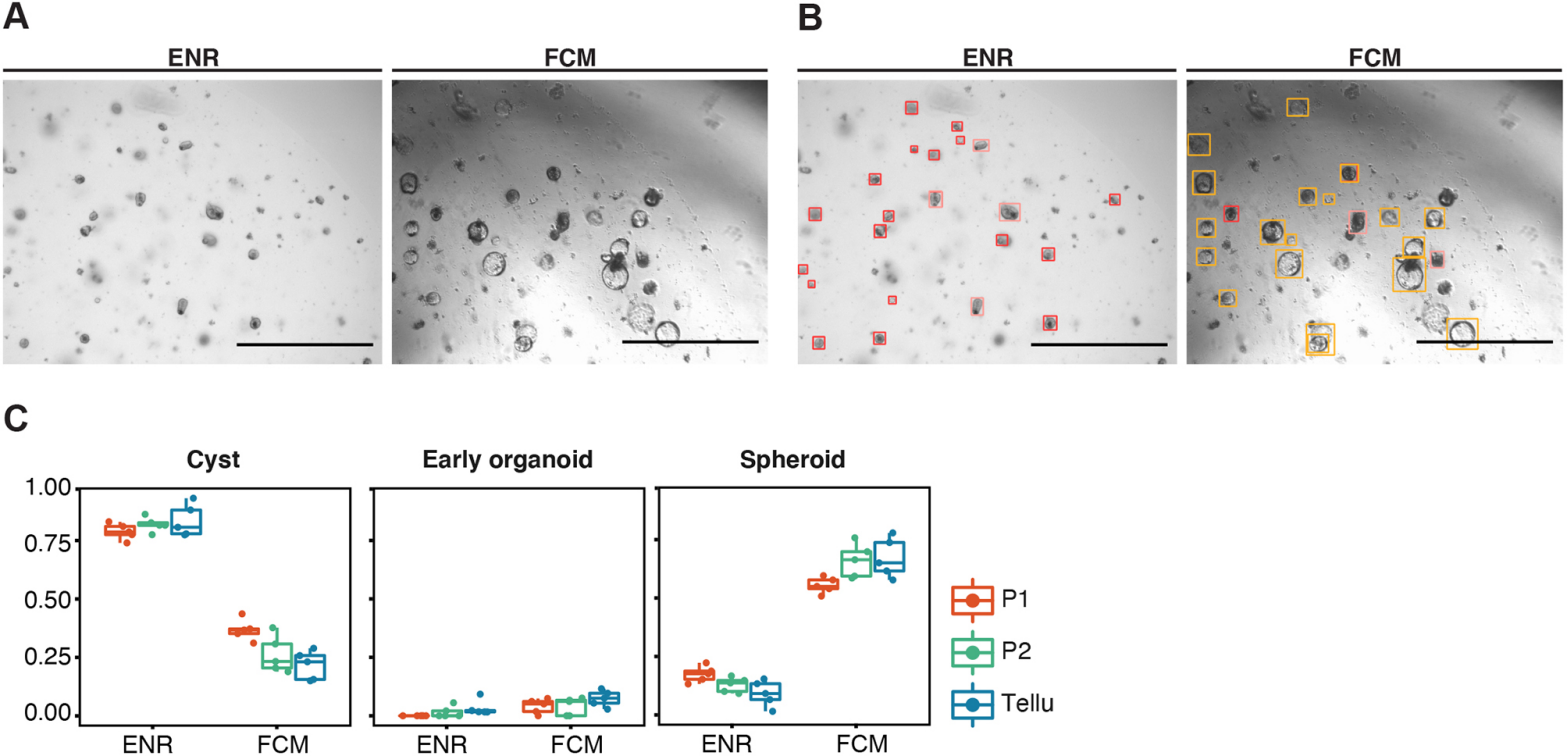
**Performance of Tellu regarding the classification of intestinal organoid under spheroid-inducing conditions.** (A) Representative images of organoids grown for 24 h under normal (ENR) or spheroid-inducing (fibroblast conditioned medium, FCM) conditions. (B) Images as shown in A but including bounding boxes surrounding objects recognised by Tellu. Scale bars: 1 mm. (C). Compared is the classification of organoids cultured in ENR or FCM medium, carried out by two experienced scientists (P1 and P2) or automated analysis (Tellu). Each data point represents a relative share of each organoid class per technical replicate (well) (*n*=255 organoids ENR, *n*=304 organoids FCM).

In a second approach, we assessed the performance of Tellu under spheroid formation-inducing conditions. To this end, classification was done by using images of intestinal crypts grown for 24 h in primary intestinal fibroblast conditioned medium (FCM) or ENR medium as control. Again, we observed very good alignment of automatic and manual classification under spheroid-inducing conditions [*cyst* P1=0.38±0.04; P2=0.27±0.08; Tellu=0.23±0.06, *spheroid* P1=0.57±0.03; P2=0.68±0.07; Tellu=0.69±0.08] (Fig. 4, FCM). At this early time, abundance of early organoids (*organoid1*) was low and late organoids (*organoid3*) were not identified in manual or automated classification. Tellu also efficiently recognised pre-tumorigenic organoids isolated from the intestines of *Apc^min^* mice or from chemically induced mouse colon tumours, which mainly grow as spheroids (Fig. S1). Thus, Tellu would also be useful when studying the growth of organoids that represent early intestinal tumorigenesis.

### Automated quantitation of relative organoid size by Tellu

**Fig. 5.**
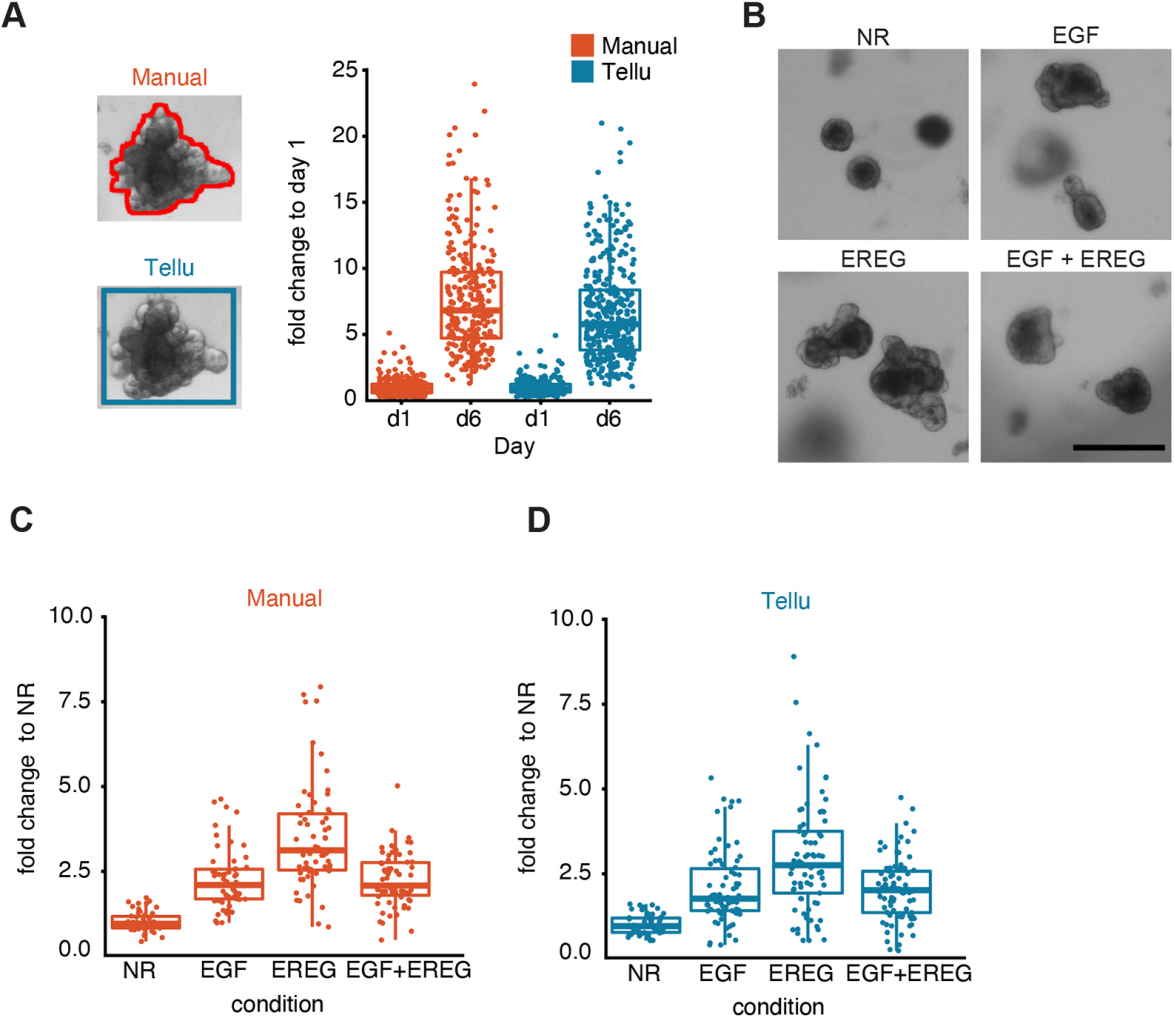
**Performance of Tellu regarding the quantification of intestinal organoid size in response to different growth factors.** (A) Representative images of manual area calculation using ImageJ (top image) or the equivalent bounding box in Tellu (bottom image). Plotted are the organoid sizes on days 1 and 6 of culture (data as shown in Fig. 3) obtained through manual (Manual) or automated (Tellu) measurements (*n*=595). (B) Representative images of organoids grown for 5 days in control medium (NR), in the presence of EGF or EREG (100 ng ml^−1^), or in the presence of EGF and EREG together (100 ng ml^−1^ each). Scale bar: 250 μm. (C,D) Comparison of manual versus Tellu quantification of organoid size (Manual: *n*=50 for NR, *n*=55 for EGF, *n*=63 for EREG, *n*=67 for EGF+EREG; Tellu: *n*=50 for NR, *n*=70 for EGF, *n*=73 for EREG, *n*=81 for EGF+EREG).

Since the detection process used by Tellu includes drawing a bounding box around each detected object in the image, we went on to assess whether the area of the bounding box can be used as a surrogate when manually calculating the size of organoids. To this end, quantification of organoid size by manual annotation of organoid boundaries with ImageJ was compared to the quantification based on the area of the bounding box around each organoid drawn by Tellu (Fig. 5A) and calculated as relative organoid growth between days 1 and 6 by using the images collected earlier (Fig. 3). Manual quantification (*n*=595) demonstrated a 7.7-fold increase in organoid size between days 1 and 6 (Fig. 5A). Quantification based on bounding boxes by Tellu showed a 6.7-fold increase (Fig. 5A), demonstrating the ability of Tellu to detect changes in organoid size. Whereas the resolution was slightly compromised due to the boxed versus accurate area approach, Tellu outperformed manual analysis with <5 min of hands-on time compared to a duration of >100 min for manual quantification.

In a second approach to evaluate the ability of Tellu to analyse organoid size in situations where differences are minor, we compared Tellu and manual quantification of organoid size in intestinal organoids that had been cultured for 5 days with epiregulin (EREG), with EGF or without any EGF-related factors (NR). This approach was based on identifying EREG as a stroma-derived growth factor potentially stimulating epithelial hyperproliferation (Ollila et al., 2018). In this experiment, alignment between automated Tellu and manual classification was robust. Both analyses showed enhanced growth in EGF-treated organoids, and further elevated growth in organoids that had been treated with EREG (Fig. 5B-D). Interestingly, combined treatment with EGF and EREG resulted in organoid size similar to that observed following treatment with EGF alone. This experiment confirms the ability of Tellu to quantitate organoid size and suggests a previously unidentified connection between signalling stimulated by different EGF receptor ligands.

## DISCUSSION

Tellu represents a new open-access tool for the automated classification of intestinal organoids. It stimulates quantitative studies on intestinal organoids by providing a low-threshold tool to analyse large numbers of organoids currently requiring significant researcher hands-on time. Based on morphology Tellu uniquely enables the classification of intestinal organoids, including the stage of crypt budding, and also provides a proxy for organoid size.

Tellu is based on the YOLO object-detection algorithm. The YOLO algorithm was chosen owing to its speed and recently developed accuracy. It divides each image into a grid containing several cells, each represented as a vector that carries the cell coordinates relative to the whole image and a confidence value indicating the presence or absence of an object. Importantly, unlike other methods, such as Fast R-CNN (Ren et al., 2017), YOLO (Redmon et al., 2016) trains on whole images at once, including cells that do not contain any objects, making YOLO less likely to mistake background for objects. In addition, each cell predicts the class of the object, represented by another vector with a length corresponding to the number of classes. Although multiple bounding boxes can be predicted, the model gives only one class for each cell. Thus, YOLO first predicts the presence or absence of an object within a defined cell and provides a confidence value for the class of each predicted object.

As compared to previously published organoid analysis tools (Kassis et al., 2019; Ostrop et al., 2020; Spiller et al., 2021), Tellu provides significant added value. In addition to being the first easy-access tool to classify organoids, the YOLOv5 object-detection algorithm makes it compatible with video formats. It can be applied in real-time experimental setups. We trained Tellu and used it on brightfield still images. Still, real-time imaging with outputs in the format of videos is also used in the field (Yang et al., 2021), and Tellu is compatible with these (Movie 1). Quantifying organoid detections in videos by using Tellu may require object tracking algorithms to avoid counting the same organoid multiple times along the *z*-axis of a *z*-stack. This feature is planned to be implemented in a future release. Another benefit of the Yolov5 network is the easy implementation in a docker repository ensuring reproducibility and customizability.

During the revision process, another organoid classification tool, OrganoID, was released (Matthews et al., 2022). OrganoID is able to count and track single organoids, including small intestinal organoids, by using a neural network based on image segmentation, making it very useful for, e.g. time-lapse experiments, where the growth of individual organoids is monitored. However, OrganoID requires a basic level of python programming skills, whereas our workflow in Colaboratory makes sure programmable skills are not required. In addition, OrganoID is optimised for pancreatic organoids, whereas Tellu was trained exclusively for the classification of small intestinal organoids.

When considering the value of a tool for organoid classification, two parameters identified for other tools stand out – speed and accuracy – where speed should not compromise accurate detection. Notably, classification by Tellu was significantly superior regarding speed and not seriously compromised for accuracy when compared to manual quantification. With Tellu, analysis only takes a few seconds per image, with very little hands-on time. Output data contain the coordinates of the bounding box that can be used in addition to classification when deriving the relative area of each organoid. To some extent, all manual quantification is – especially in terms of classification – subjective and prone to bias as well as variation between experiments. Tellu performs consistently across large datasets without bias and, thus, is superior to manual analyses regarding reproducibility.

However, despite its numerous advantages, Tellu also has limitations. In dense organoid cultures where multiple organoids overlap, Tellu – as well as manual counters – struggles to distinguish organoids from each other. The organoid area calculation by Tellu is an approximation, as the bounding box does not account for the complex surface architecture of the organoids. However, these limitations are predictable and reproducible, and therefore do not compromise the value of Tellu in providing a quantitative, reproducible tool to classify intestinal organoids.

## METHODS

### Crypt isolation and organoid culture

After removing the entire small intestine from the mouse, the mesentery was removed, and the intestine was flushed with cold PBS using a syringe and a 21-gauge needle. The intestine was cut open longitudinally and mucus removed by washing, i.e. immersing and shaking, the tissue three times in cold PBS. Thereafter, the tissue was minced into 3 mm fragments, placed horizontally in a 50 ml Falcon tube with 20-30 ml 10 mM EDTA in PBS, and left on ice for 1.45 h. During the first 45 min, the EDTA/PBS solution was changed every 15 min, after gently shaking the tube for 15 s and letting the tissue pieces sink before changing the solution. After incubation, crypts were detached by vigorous shaking for 20 s with two shakes per second. Crypts were isolated from villi and tissue pieces by filtering through a 70 µm strainer, pelleted by spinning the supernatant for 2 min at 200 rpm, washed with PBS, and collected as before. Crypts were resuspended in basal medium (DMEM:F12 medium (12634010, Gibco) supplemented with 1 M HEPES buffer pH 7,4 (H0887, Sigma), 1×Glutamax (35050061, Gibco), 1×Pen-strep (P4333, Sigma), 1×N-2 Supplement (17502001, Gibco) and 1× B-27 Supplement (17504044, Gibco) complemented with 50 ng ml^−1^ of EGF (PMG8041, Gibco), 100 ng ml^−1^ of Noggin (250-38, Peprotech), 250 ng ml^−1^ of R-spondin 1 (3474-RS, R&D systems), 1 µM of N-acetylcysteine (A-5131, Sigma) and 10 µM of the ROCK1/2 inhibitor Y-27632 (Y0503, Sigma; used for first 2 days of culture), and mixed 1:3 ratio with Matrigel (356231, Corning) before plating 12 µl domes to 48-well plates. Matrigel domes were overlaid with ENR medium. Organoid medium was changed every two days. In experiments comparing the effects of the EGF receptor ligands EGF (R&D Systems 236-EG) and EREG (R&D Systems 968-EP), both ligands were used at 100 ng ml^−1^.

### Fibroblast isolation and collection of conditioned medium

Primary intestinal fibroblasts were isolated using an adapted version of a previously published protocol (Stzepourginski et al., 2015). Mouse small intestine was washed with PBS, ripped open and cut into 1 cm fragments. Tissue fragments were incubated in PBS containing EDTA (5 mM) and DTT (1 mM) for 20 min, and placed horizontally in a rotating (250 rpm) incubator at 37°C. EDTA solution was removed by washing tissue pieces three times with cold PBS by vigorously shaking for 15 s by hand, followed by foam removal and replacement of PBS between shakes. After the washes, the pieces were placed in PBS and minced thoroughly with a scalpel. Tissue pieces were then placed in a collagenase digestion solution [2 mg ml^−1^ of collagenase type II and IV (Thermo Fisher Scientific) at a ratio of 1:1 dissolved in PBS containing 2% FBS] and horizontally rotated at 250 rpm for 20 min at 37°C. Cold fibroblast growth medium (DMEM+20% FBS+1% penicillin/streptomycin+1% L-glutamine) was added to stop collagenase activity. Single cells were released from the digestion suspension by trituration, and cells were pelleted by centrifugation (10 min, 1300 rpm) and resuspended in growth medium at 37°C. The suspension was plated on a Petri dish and cells were allowed to attach for 1 hour at 37°C. Thereafter, cells were washed three times with PBS to remove debris and unattached cells, and fresh medium was added. To collect fibroblast conditioned medium, 1×10^5^ intestinal fibroblasts were grown for 24 h in 6 cm plates in DMEM supplemented with 10% FBS. Medium was then changed to organoid basal medium for another 24 h, after which it was collected and centrifuged (5 min at 1100 rpm) to remove debris. Of this fibroblast conditioned medium, 350 µl were used per organoid-containing Matrigel dome. Organoids were imaged after 48 h.

### Intestinal *Apc* mutant tumour-derived organoids

To culture tumorigenic intestinal organoids, small intestinal crypts were collected from 15-17-week-old tumour-bearing *Apc*^min^ mice (JAX strain 002020), used to model the early stages of human colorectal cancer development (Su et al., 1992). The crypts were mixed with ENR medium and plated at 1:3 ratio with Matrigel as described before. After 2 days, the WNT agonist RSPO1 was removed from growth medium to select biallelic *Apc* mutant organoids that survive without exogenous WNT activation as described (Langlands et al., 2018). *Apc* mutant organoids were passaged and harvested using an ice-cold organoid harvesting solution (3700-100-01, R&D Systems) for at least 30 min. Matrigel domes were then scraped from the plate, pipetted 10× before collecting into a tube, and centrifuged at 300 ***g*** for 5 min. Then organoids were mechanically dissociated by pipetting up and down 40-50 times, and mixed with Matrigel as mentioned above.

### Chemically induced colon tumour-derived organoids

By using two 7-day rounds of 2.5% DSS in drinking water, colonic tumours were induced following the azoxymethane–dextran sodium sulphate (AOM–DSS) protocol as described (Thaker et al., 2012). Used were male wild-type mice aged 10-12 weeks with a mixed genetic background. Animals were housed at the University of Helsinki Laboratory Animal Center following national and European legislation. Mice were euthanised 10 weeks after injection of AOM, the tumours of the distal colon cut into small pieces with a scalpel and placed in 5 ml of DMEM supplemented with 2.5 mg ml^−1^ of collagenase 4 (17104019, Gibco) and 0.2 mg ml^−1^ of DNase 1 (10104159001, Roche). Tumour pieces were shaken (130 rpm) at 37°C for 40 min and triturated 10× with a 5 ml pipet to dissociate the cells. Supernatants were collected and spun down at 1300 rpm for 5 min. Pellets were resuspended in ENR medium with 2.5 µM CHIR99021 (SML1046, Sigma) and mixed with Matrigel. After the first two passages, organoids were grown in EN medium, followed by culturing in Noggin only (N) medium after the second passage.

### Dataset creation

By using an EVOS FL microscope (Thermo Fisher) with a 4× objective, a total of 840 transmitted-light microscopy images were taken of intestinal organoids grown at different densities and obtained during different culture stages. Images were manually annotated using the python library *labelImg* and exported for the *Yolo* format as text files. The four categories of organoids in the text files were coded with integers, i.e. 0 for *organoid0*, 1 for *organoid1*, 2 for *organoid3* and 3 for *spheroid*. (Fig. 1B). The dataset images were split into a training and a validation set consisting of 756 and 84 images, respectively, by using the python library module *splitfolders*. The annotated dataset has been registered at zenodo under doi:10.5281/zenodo.6768583.

### Model training

The Yolov5 (DOI:10.5281/zenodo.3908559) state-of-the-art object detector was trained for 100 epochs with an image batch size (i.e. the number of images the algorithm works at the same time) of 28 and using the model yolov5 s.pt as pre-weights. The code was run in an Nvidia Tesla k8 graphics card from the Colaboratory research environment.

### Organoid quantification

For automated quantification, images were analysed by Tellu using the code in the Jupyter notebook (https://colab.research.google.com/drive/1j-jutA52LlPuB4WGBDaoAhimqXC3xguX?usp=sharing) that is accessible through the GitHub repository (https://github.com/evdomene/tellervo). As an output, Tellu provides a text file for each analysed image, each row representing a detected object (organoid). The text file includes information on the organoid class, bounding box coordinates and confidence score. Files were tidied into a single file with all detections and metadata information, and further summarised into different condition groups by using R packages tidyverse and ggpubr for plotting.

Two independent researchers counted each organoid class in each image for manual quantification and summarised them by wells (four images per well). Further quantification and plots were done with R packages tidyverse and ggpubr (see above).

## Supplementary Material

10.1242/dmm.049756_sup1 Supplementary information

## References

[DMM049756C1] Almeqdadi, M., Mana, M. D., Roper, J. and Yilmaz, Ö. H. (2019). Gut organoids: mini-tissues in culture to study intestinal physiology and disease. *Am. J. Physiol. Cell Physiol.* 317, C405-C419. 10.1152/ajpcell.00300.2017 31216420 PMC6766612

[DMM049756C2] Farin, H. F., Van Es, J. H. and Clevers, H. (2012). Redundant sources of Wnt regulate intestinal stem cells and promote formation of Paneth cells. *Gastroenterology* 143, 1518-1529.e7. 10.1053/j.gastro.2012.08.031 22922422

[DMM049756C3] Fordham, R. P., Yui, S., Hannan, N. R. F., Soendergaard, C., Madgwick, A., Schweiger, P. J., Nielsen, O. H., Vallier, L., Pedersen, R. A., Nakamura, T. et al. (2013). Transplantation of expanded fetal intestinal progenitors contributes to colon regeneration after injury. *Cell Stem Cell* 13, 734-744. 10.1016/j.stem.2013.09.015 24139758 PMC3858813

[DMM049756C5] Guiu, J., Hannezo, E., Yui, S., Demharter, S., Ulyanchenko, S., Maimets, M., Jørgensen, A., Perlman, S., Lundvall, L., Mamsen, L. S. et al. (2019). Tracing the origin of adult intestinal stem cells. *Nature* 570, 107-111. 10.1038/s41586-019-1212-5 31092921 PMC6986928

[DMM049756C6] Kassis, T., Hernandez-Gordillo, V., Langer, R. and Griffith, L. G. (2019). OrgaQuant: human intestinal organoid localization and quantification using deep convolutional neural networks. *Sci. Rep.* 9, 12479. 10.1038/s41598-019-48874-y 31462669 PMC6713702

[DMM049756C7] Langlands, A. J., Carroll, T. D., Chen, Y. and Näthke, I. (2018). Chir99021 and Valproic acid reduce the proliferative advantage of Apc mutant cells. *Cell Death Dis.* 9, 255. 10.1038/s41419-017-0199-9 29449562 PMC5833359

[DMM049756C8] Matthews, J. M., Schuster, B., Kashaf, S. S., Liu, P., Ben-Yishay, R., Ishay-Ronen, D., Izumchenko, E., Shen, L., Weber, C. R., Bielski, M. et al. (2022). OrganoID: A versatile deep learning platform for tracking and analysis of single-organoid dynamics. *PLoS Comput. Biol.* 18, e1010584. 10.1371/journal.pcbi.1010584 36350878 PMC9645660

[DMM049756C9] Nusse, Y. M., Savage, A. K., Marangoni, P., Rosendahl-Huber, A. K. M., Landman, T. A., de Sauvage, F. J., Locksley, R. M. and Klein, O. D. (2018). Parasitic helminths induce fetal-like reversion in the intestinal stem cell niche. *Nature* 559, 109-113. 10.1038/s41586-018-0257-1 29950724 PMC6042247

[DMM049756C10] Ollila, S., Domènech-Moreno, E., Laajanen, K., Wong, I. P., Tripathi, S., Pentinmikko, N., Gao, Y., Yan, Y., Niemelä, E. H., Wang, T. C. et al. (2018). Stromal Lkb1 deficiency leads to gastrointestinal tumorigenesis involving the IL-11-JAK/STAT3 pathway. *J. Clin. Invest.* 128, 402-414. 10.1172/JCI93597 29202476 PMC5749537

[DMM049756C11] Ostrop, J., Zwiggelaar, R. T., Terndrup Pedersen, M., Gerbe, F., Bösl, K., Lindholm, H. T., Díez-Sánchez, A., Parmar, N., Radetzki, S., von Kries, J. P. et al. (2020). A semi-automated organoid screening method demonstrates epigenetic control of intestinal epithelial differentiation. *Front. Cell Dev. Biol.* 8, 618552. 10.3389/fcell.2020.618552 33575256 PMC7872100

[DMM049756C12] Redmon, J., Divvala, S., Girshick, R. and Farhadi, A. (2016). You Only Look Once: Unified, Real-Time Object Detection. IEEE Conference on Computer Vision and Pattern Recognition (CVPR), Las Vegas, NV, USA, 2016, pp. 779-788. 10.1109/CVPR.2016.91.

[DMM049756C4] Ren, S., He, K., Girshick, R. and Sun, J. (2017). Faster R-CNN: Towards Real-Time Object Detection with Region Proposal Networks. *IEEE Trans. Pattern Anal. Mach. Intell.* 39, 1137-1149. 10.1109/TPAMI.2016.2577031 27295650

[DMM049756C13] Sato, T., Vries, R. G., Snippert, H. J., van de Wetering, M., Barker, N., Stange, D. E., van Es, J. H., Abo, A., Kujala, P., Peters, P. J. et al. (2009). Single Lgr5 stem cells build crypt-villus structures in vitro without a mesenchymal niche. *Nature* 459, 262-265. 10.1038/nature07935 19329995

[DMM049756C14] Sato, T., Stange, D. E., Ferrante, M., Vries, R. G. J., Van Es, J. H., Van den Brink, S., Van Houdt, W. J., Pronk, A., Van Gorp, J., Siersema, P. D. et al. (2011). Long-term expansion of epithelial organoids from human colon, adenoma, adenocarcinoma, and Barrett's epithelium. *Gastroenterology* 141, 1762-1772. 10.1053/j.gastro.2011.07.050 21889923

[DMM049756C15] Serra, D., Mayr, U., Boni, A., Lukonin, I., Rempfler, M., Challet Meylan, L., Stadler, M. B., Strnad, P., Papasaikas, P., Vischi, D. et al. (2019). Self-organization and symmetry breaking in intestinal organoid development. *Nature* 569, 66-72. 10.1038/s41586-019-1146-y 31019299 PMC6544541

[DMM049756C16] Spiller, E. R., Ung, N., Kim, S., Patsch, K., Lau, R., Strelez, C., Doshi, C., Choung, S., Choi, B., Juarez Rosales, E. F. et al. (2021). Imaging-based machine learning analysis of patient-derived tumor organoid drug response. *Frontiers in Oncology* 11, 771173. 10.3389/fonc.2021.771173 34993134 PMC8724556

[DMM049756C17] Stzepourginski, I., Eberl, G. and Peduto, L. (2015). An optimized protocol for isolating lymphoid stromal cells from the intestinal lamina propria. *J. Immunol. Methods* 421, 14-19. 10.1016/j.jim.2014.11.013 25599879

[DMM049756C18] Su, L. K., Kinzler, K. W., Vogelstein, B., Preisinger, A. C., Moser, A. R., Luongo, C., Gould, K. A. and Dove, W. F. (1992). Multiple intestinal neoplasia caused by a mutation in the murine homolog of the APC gene. *Science* 256, 668-670. 10.1126/science.1350108 1350108

[DMM049756C19] Thaker, A. I., Shaker, A., Rao, M. S. and Ciorba, M. A. (2012). Modeling colitis-associated cancer with azoxymethane (AOM) and dextran sulfate sodium (DSS). *J. Vis. Exp.* 67, 4100. 10.3791/4100 PMC3490277 22990604

[DMM049756C20] Yang, Q., Xue, S.-L., Chan, C. J., Rempfler, M., Vischi, D., Maurer-Gutierrez, F., Hiiragi, T., Hannezo, E. and Liberali, P. (2021). Cell fate coordinates mechano-osmotic forces in intestinal crypt formation. *Nat. Cell Biol.* 23, 733-744. 10.1038/s41556-021-00700-2 34155381 PMC7611267

