# Supplementary material for: Tellu – an object-detector algorithm for automatic classification of intestinal organoids
Source: Dis Model Mech. 2023 Mar 13;16(3):dmm049756. doi: 10.1242/dmm.049756 (PMC10067441; doi:10.1242/dmm.049756)
Supplement: Supplementary information [file dmm-16-049756-s1.pdf]

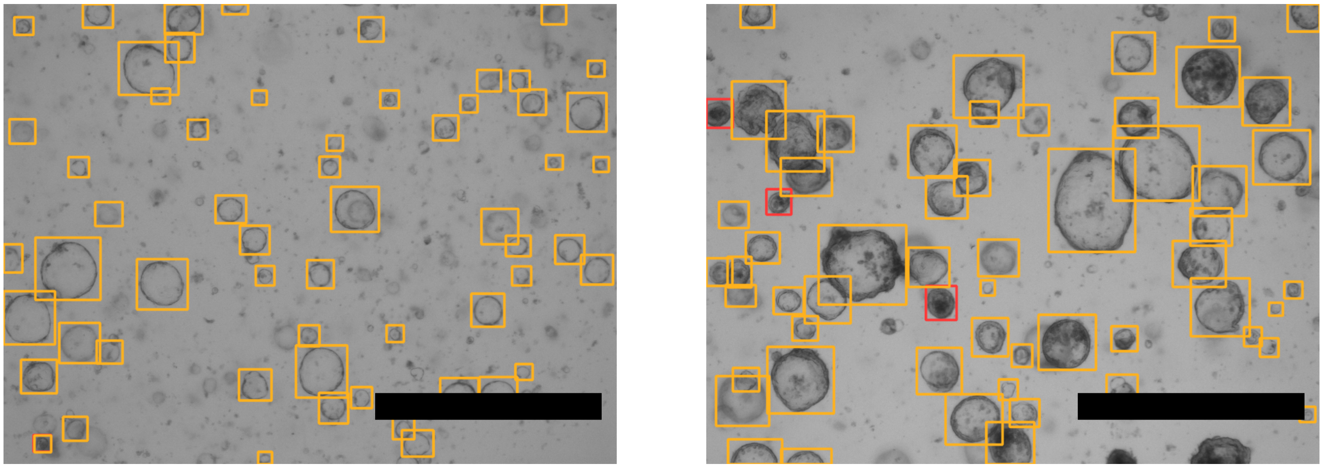

Fig. S1. Representative images of tumour-derived organoids from *APC*^{min} mice and AOM-DSS treated mice.

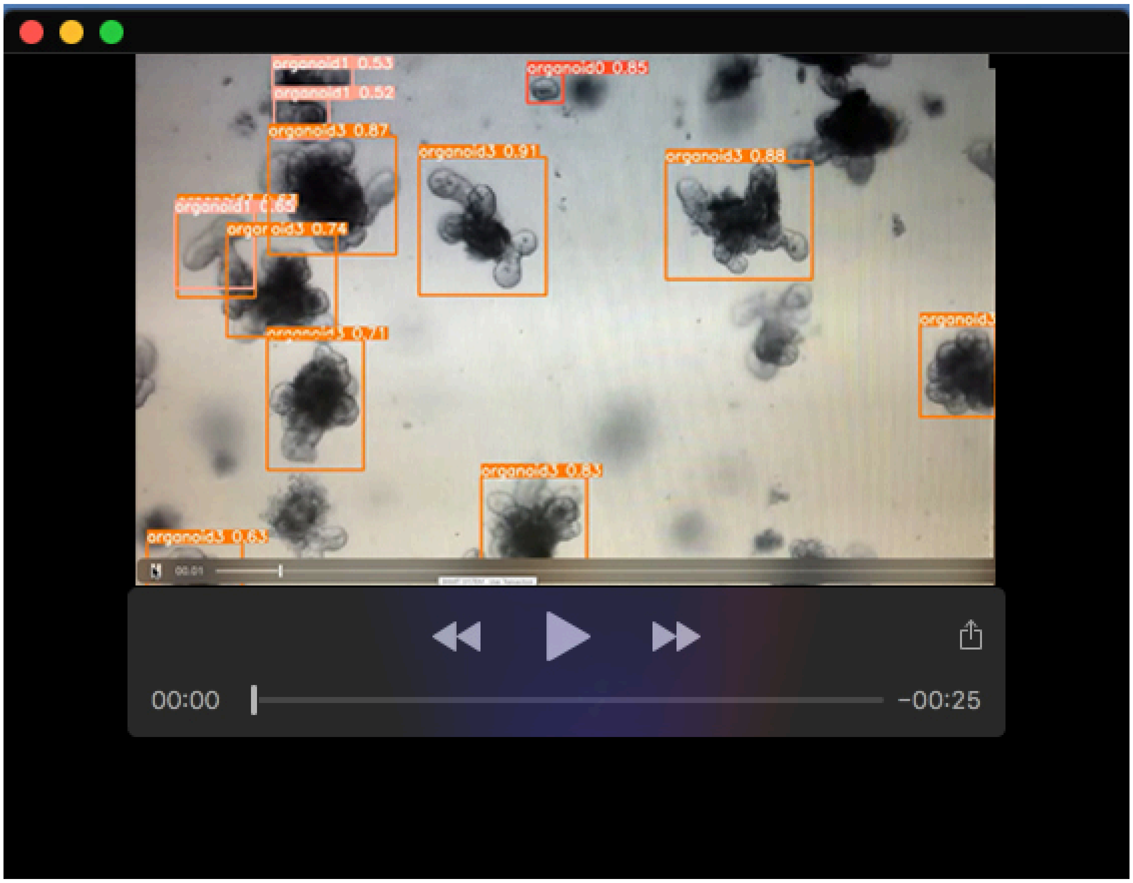

Movie 1. Tellu classifies organoids in video format, making it compatible with real-time imaging experiments.
